# Wand Stretching Exercise Decreases Abdominal Obesity Among Adults With High Body Mass Index Without Altering Fat Oxidation

**DOI:** 10.3389/fphys.2020.565573

**Published:** 2020-10-29

**Authors:** Punnee Puengsuwan, Chia-Hua Kuo, Rungchai Chaunchaiyakul, Ratanavadee Nanagara, Naruemon Leelayuwat

**Affiliations:** ^1^School of Physical Therapy, Faculty of Associated Medical Sciences, Khon Kaen University, Khon Kaen, Thailand; ^2^Laboratory of Exercise Biochemistry, University of Taipei, Taipei, Taiwan; ^3^College of Sports Science and Technology, Mahidol University, Nakhon Pathom, Thailand; ^4^Department of Medicine, Faculty of Medicine, Khon Kaen University, Khon Kaen, Thailand; ^5^Department of Physiology, Faculty of Medicine, Exercise and Sport Sciences Development and Research Group, Khon Kaen University, Khon Kaen, Thailand; ^6^Graduate School, Khon Kaen University, Khon Kaen, Thailand

**Keywords:** aerobic exercise, cardiovascular risk factors, abdominal obesity, waist circumference, energy expenditure

## Abstract

**Rationale:**

We designed a wand-based muscle stretching (WE) exercise program, which has become increasingly popular in physical therapy and has been used for elderly patients with adhesive capsulitis. However, studies regarding the effects of WE training on abdominal obesity and measures of cardiovascular risk factors among overweight/obese adults aged ≥55 years are rare.

**Purpose:**

The objective of this study is to evaluate the effects of a 15-week wand stretching exercise program on waist circumference and cardiovascular risk factors in sedentary adults aged 55–70 years.

**Methods:**

A total of 124 participants were randomly assigned to either participate in wand stretching exercise (WE) over a 15-week period or a control group (*n* = 62 each). Sixty participants in the WE group (26 overweight and 34 obese) and 51 in the control group (29 overweight and 22 obese) completed the study. The WE program included wand-assisted muscle stretching exercise on both the upper body and lower body for 40 min per day, 5 days per week, whereas the control group maintained their sedentary lifestyle.

**Results:**

No significant improvements were observed in plasma glucose, insulin, and the homeostatic model assessment of insulin resistance (HOMA-IR) after exercise training. Compared with the control group, the WE group had more significant reductions in waist circumference among participants with a body mass index (BMI) < 25 kg/m^2^ (−2.6 cm, 95% CI: −4.19 to −0.97 cm, *d* = 0.48) and BMI > 25 kg/m^2^ (−2.5 cm, 95% CI: −4.1 to −0.9 cm, *d* = 0.59) (both *P* < 0.01). Furthermore, within groups, a significant increase in % fat free mass was observed after WE training. The basal metabolic rate was slightly increased, but the fat oxidation rate remained unaltered in the WE group. Improvements in low-density lipoprotein cholesterol to high-density lipoprotein cholesterol were minimal after WE. Significant reductions in high-sensitivity C-reactive protein were observed after WE only for participants with a BMI <25 kg/m^2^.

**Conclusion:**

The results suggest redistribution of a carbon source from the abdominal region to challenged skeletal muscle, following prolonged WE training. This abdominal fat reducing outcome of the WE is unlikely to be associated with fatty acid oxidation.

## Introduction

According to the Asian-Pacific cutoff points, overweight is classified as a body mass index (BMI) between 23.0 and 24.9 kg/m^2^, and obesity, higher than 25 kg/m^2^ (World Health Organization. Regional Office for the Western Pacific, 2000). The correlation between the incidence of cardiovascular disease (CVD) and that of abdominal fat has been reported, particularly among obese subjects (Rexrode et al., 2001; Onat et al., 2004; Nicklas et al., 2006). Other cardiovascular risk factors (CVRF) among obese persons include insulin resistance (Kouvari et al., 2019), dyslipidemia (Syed et al., 2009; Jackisch et al., 2018), and a reduced basal metabolic rate (BMR) (Bhopal and Rafnsson, 2009). The risk of CVD is associated with persistent low-grade systemic inflammation, as indicated by elevated high-sensitivity C-reactive protein (hsCRP) (Pai et al., 2004).

Regular moderate-intensity endurance exercise has been recommended to minimize CVRF (American College of Sports Medicine (ACSM), 2000), including abdominal fat (Rashti et al., 2019; Jiang et al., 2020) and low-density lipoprotein (LDL) (Izquierdo-Porrera et al., 2000), and increase insulin sensitivity, high-density lipoprotein (HDL), and BMR (Morio et al., 1998). The effects of exercise training in lowering persistent systemic inflammation has been confirmed by reports of reduced hsCRP levels (Kohut et al., 2006). However, the beneficial effects of aerobic training on improving insulin sensitivity and glycemic control diminishes with age and is particularly ineffective for those aged ≥55 years (Short et al., 2003). Strength training remains an effective intervention for metabolic outcomes for this age group (Cauza et al., 2005), suggesting that stretching forms of muscle contraction that poses less of a challenge to the cardiopulmonary system is beneficial for metabolic improvement among older individuals. However, the exercise modality employed in the present study is not recommended for those with limited mobility in that age group. In this study, we designed a wand-based muscle stretching exercise (WE) program, which has become increasingly popular in physical therapy and has been used for elderly patients with adhesive capsulitis (frozen shoulder). Importantly, WE is the combination of flexibility, balance, and endurance, which has been recommended for its positive outcomes for individuals (Puengsuwan et al., 2008). With simple and slow movements in all directions, WE will possibly be another handy home-based exercise tool for health promotion in older people. The effects of this mode of exercise on abdominal obesity and CVRF have not been previously evaluated. Obese individuals often developed systemic inflammation with high hsCRP levels, which causes limited joint mobility (Anson et al., 2018). However, studies regarding the effects of WE training on abdominal obesity and measures of CVRF among overweight/obese adults aged ≥55 years are rare. Therefore, in this study, we aimed to investigate whether WE training is an effective intervention that can reduce CVRF for overweight and obese adults. We hypothesized that reductions in waist circumference, CVRF measures, BMR, and hsCRP would be evident among overweight/obese individuals aged ≥55 years, after prolonged WE training.

## Materials and Methods

### Participants

A total of 124 participants, from the urban area of Khon Kaen, Thailand, gave full informed consent after verbal and written explanations of the details of the study. They were recruited via poster and personal contact. The inclusion criteria included those with: (a) a healthy sedentary lifestyle (exercised <30 min twice per week during the previous 12 months); (b) aged between 55 and 70 years; (c) BMI ≥ 23 kg/m^2^; and (d) menopause. Participants were divided into two groups; overweight and obese, based on the cutoff point of a BMI of 25 kg/m^2^. In particular, Thai participants with BMI >25 kg/m^2^ were classified as obese (World Health Organization. Regional Office for the Western Pacific, 2000). Exclusion criteria included non-obesity with normal blood glucose and lipid levels, and blood pressure within the normal range (Grundy et al., 2005) in addition to impaired mobility, hepatic, and renal functions. Participants who regularly engaged in exercise more than twice/week within the previous 3 months were also excluded.

**FIGURE 1:**
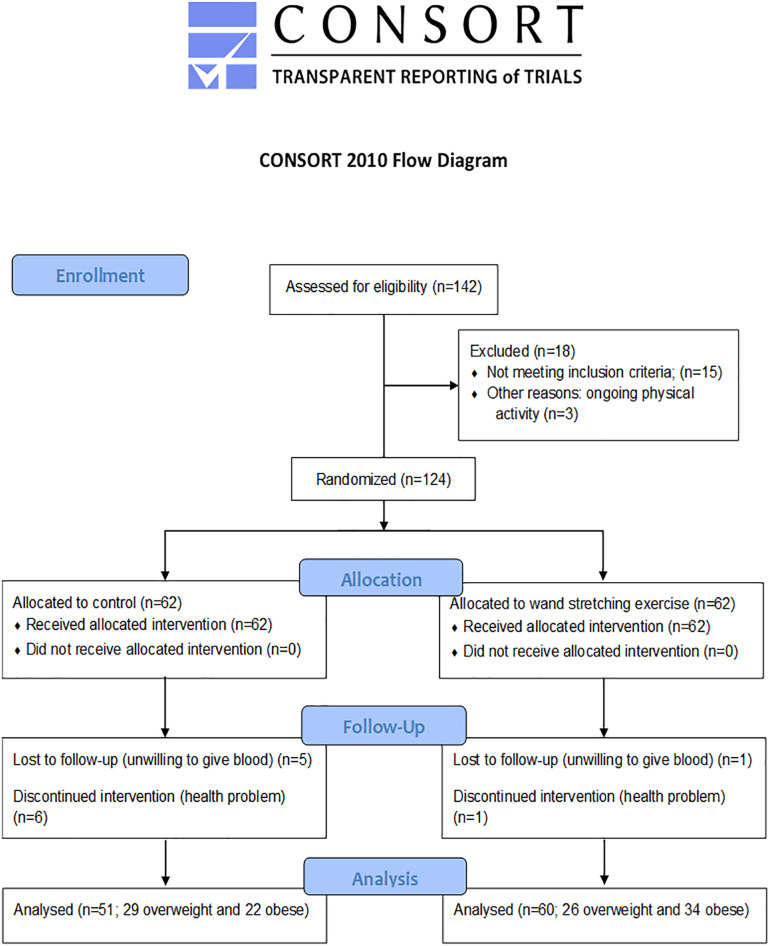
Flow diagram of this study.

All participants completed a routine medical examination comprising a health questionnaire, physical examination, blood chemistry assessment, and 12-lead electrocardiograph. They were subsequently randomly allocated to either the training or control cohorts with the aid of a computer-generated randomization list that assigned 71 participants to each cohort. This study was approved by the Ethics Committee of Khon Kaen University (HE480102) and conducted in accordance with the 1964 Declaration of Helsinki. A flow diagram of the study as outlined in the Consolidated Standards of Report Trials (CONSORT) statement is shown in Figure 1.

### Experimental Design and Protocol

A randomized, single-blind, parallel group, two-arm clinical trial was conducted between February 2015 and April 2016 at the School of Physical Therapy, Faculty of Associated Medical Sciences and Department of Physiology, Faculty of Medicine, Khon Kaen University. All participants were screened via medical history, physical examination, electrocardiography, and blood sampling for routine blood chemistry and hematology. Participants were then randomly assigned either to a 15-week period of wand exercise (WE group) or continuation of a sedentary lifestyle (control group) (Figure 1).

The WE training consisted of stretching and aerobic exercise 40 min a day, 5 days a week, whereas the control group engaged in their routine activities of daily living without any other regular exercise (<2 days per week). Adherence to the WE was monitored at the laboratory during week 2 and tracked via monthly telephone communication. Before and after 15 weeks, parameters were measured. After a 12-h fast on the day of measurement, participants arrived at the laboratory at 7.30 am and anthropometry and body composition were measured. They then assumed a supine position on a bed and 5-min expired air was collected for analysis of the BMR.

Sample size was calculated using the mean and standard deviation (SD) based on the study of Puengsuwan et al. (2008). Power of the statistical tests was calculated based on different means of abdominal obesity between two dependent groups, using the STATA Version 10 software (StataCorp LLC, United States). At least 62 participants (including 20% dropouts) in each group were required to show significant differences at a 5% significance level, and Cohen’s *d* effect size was 0.23. The power (1 - β) was 0.83.

### Wand Exercise Program

**FIGURE 2:**
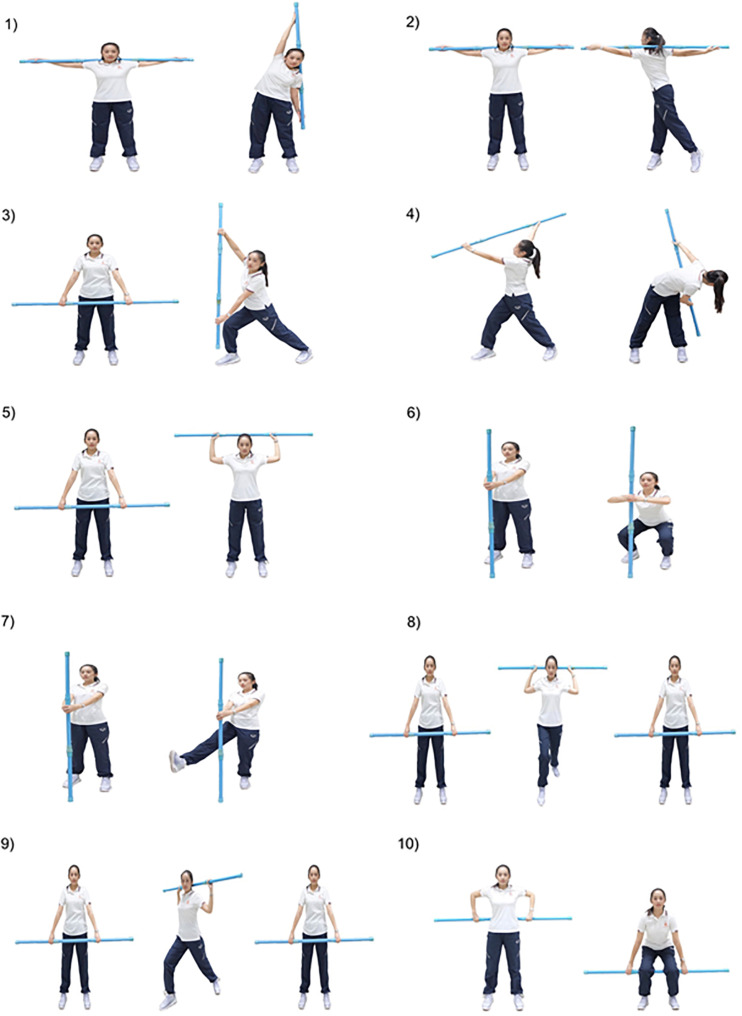
Wand stretching exercise.

The WE program comprised a series of exercises, which were all performed in the standing position, while holding a 770 g wand. The exercise included 10 movements of the upper and lower body (Figure 2). The movements of the upper body comprised movements of the upper arms and trunk around the waist, including flexion and extension, lateral flexion and rotation of the trunk, and flexion, extension, adduction, abduction, and diagonal flexion and extension of the shoulders. The movements of the lower body comprised flexion, extension, abduction, adduction, and rotation of the hip, and flexion and extension of the knee joints (Supplementary Figure 1).

Participants in the training cohort were permitted to adjust the length of the wand to suit their height and arm span. Participants successfully performed all movements of the WE on the first visit. Subsequently, they were asked to repeat these exercises as their home-based program for the next 15 weeks via the teaching media of a video recording. Throughout the 15 weeks, participants in the control cohort did not engage in any regular exercise (<2 days per week). They maintained their usual levels of sedentary physical activity and caloric intake.

### Standard Biosecurity and Institutional Safety Procedures

To be familiar with and prevent injury from the WE, they performed the WE during a 20-min session on each of 3 days for the first 3 weeks. After the first 3 weeks, all participants in the training cohort were asked to return to the laboratory for reassessment of the program and adjustment. Then, they performed the WE training by two 20-min sessions per day, 5 days per week for the next 12 weeks. A telephone call was made to each participant every month during the study period to verify their compliance, to report whether there was any injury according to the exercise, and encourage them to maintain their usual levels of daily physical activity (over and above the WE program).

### Outcome Measurements

#### Anthropometry and Body Composition

The weights and heights of all participants were recorded and the BMI was later computed. The waist circumference (WC) was measured midway between the costal margin and the iliac crest at the end of inspiration. Hip circumference (HC) was measured as the greatest value over the buttocks. Fat mass was measured indirectly using the skinfold thickness method. Specifically, skinfold thickness was measured at four sites- the triceps, biceps, subscapular, and suprailiac crest, using a caliper. These measurements were then used to evaluate body fat, applying the equations estimated by Durnin and Womersley (1974). Fat free mass was then calculated based on the BM and fat mass. The coefficients of variation which is widely used to express the precision and repeatability of the measurements of WC, HC, and skinfold thickness were 2.1, 3.2, and 4.8%, respectively.

### Basal Metabolic Rate

The BMR was measured, using the technique of indirect calorimetry (Cortex-MetaMax 3x Series, Germany). Calibrations were done prior to each measurement using known gas concentrations and a 3.0-L gas analyzer syringe. Participants were required to refrain from any strenuous exercise for at least 24 h prior to the test and after a night’s sleep, and were subjected to a fast (including no breakfast). The BMR was measured via a breathing mask over a period of 30 min, while participants rested quietly in a supine position in an isolated room maintained between 21 and 26°C. Oxygen consumption *V̇*O_2_, (L/min) and carbon dioxide production *V̇*CO_2_ (L/min) rates were recorded and used to calculate the BMR according to the following formula developed by Weir (1949):

(1) $$\mathrm{BMR}\left(\mathrm{Cal}/\mathrm{day}\right)=\left[\left(3.9\times \dot{V}O_{2}\right)+\left(1.1\times \dot{V}CO_{2}\right)\right]\times 1.44\times 4.184\,\left(\mathrm{kJ}/\mathrm{day}\right)$$

### Fat Oxidation Rate

Fat oxidation rate (g/min) was calculated from the expired gas *V̇*O_2_ and *V̇*CO_2_, according to the following equation developed by Peronnet and Massicotte (1991) (non-protein respiratory quotient):

(2) $$\mathrm{Fat}\,\mathrm{oxidation}\,\mathrm{rate}\,\left(\text{g}/\text{min}\right)=\left(1.695\times \dot{V}\,{\text{O}}_{2}\right)\,-\,\left(1.701\times \dot{V}\,{\text{CO}}_{2}\right)$$

### Blood Chemistry Analysis

Venous blood samples were obtained after a 12-h overnight fast and analyzed using standard automated laboratory methods at the Laboratory of Srinagarind Hospital, Faculty of Medicine, Khon Kaen University, Thailand. The measurements obtained included fasting blood glucose, serum total cholesterol (TC), HDL and LDL (Roche Integra 800, Basel, Switzerland), and hsCRP concentrations (BN ProSpec, Dade Behring Marburg GmbH, United States). In addition, serum insulin concentration was measured using radioimmunoassay with a commercial kit (I^125^/RIA) from MP Biomedicals, LLC (Irvine, CA, United States). Insulin sensitivity was determined by the homeostatic model assessment of insulin resistance (HOMA-IR) technique described by Matthews et al. (1985), whereby:

HOMA-IR=glucose(mmol/L))×insulin(μU/mL)/22.5.

Compliance with the target workloads and number of sessions was > 90%.

### Dietary Intake and Physical Activity

All participants were requested to record all dietary intake and physical activities for 3 days (2 weekdays and 1 weekend day) using questionnaires. The dietary intake record was analyzed using a computerized food composition database, the INMUCAL program (Mahidol University, Thailand, 2001) and provided values from dietary components of the participants’ meals (carbohydrate, fat, and protein). In addition, participants completed a questionnaire to record the duration and frequency of activities, including occupational work, household activities, sports and leisure activities, and sleeping. Every participant in both cohorts was contacted once every month via telephone calls to verify whether they were maintaining their daily dietary intake and physical activities, including WE records.

### Statistical Analysis

The data were tested for normal distribution using the Shapiro–Wilk test. Differences between treatments (control and WE group) and within treatment (before and after experiments) were tested using repeated measures analysis of variance (ANOVA). Bonferroni’s *post hoc* test was used to define the pairs with significant differences. A last-observation-carried-forward analysis approach was used to consider the fact that the original 124 participants recruited was reduced to 111 by the end of the study [60 in the WE group (26 overweight and 34 obese) and 51 in the control group (29 overweight and 22 obese)]. Data analysis was performed using STATA: Software for Statistics and Data Science (Version 10)^1^. Statistical significance was achieved with 80% power (β) and a *p*-value <0.05. Hedges’ *g* was calculated as a measurement of effect size (Rosenthal, 1994). Measurements were expressed as means ± SE for normally distributed data, unless otherwise stated.

## Results

Of the 124 participants in the study (62 participants in each group), 60 in the WE group (26 overweight and 34 obese) and 51 in the control group (29 overweight and 22 obese) were fully compliant with the study. In the WE group; two participants withdrew from the study. One withdrew because of health problems unrelated to the exercise training program and the other was unwilling to permit blood sampling. In the control group, 11 participants withdrew from the study because of personal reasons (i.e., unwillingness to provide blood samples and health problems unrelated to the exercise training program).

No differences were noted between the groups in baseline measurements obtained by analysis of anthropometry, respiratory parameters, and blood chemistry. This indicated that the training and control cohorts had similar physiological characteristics facilitated through randomization (Table 1). No significant differences were noted in baseline daily dietary intake and energy expenditure between the training and control cohorts (*P* < 0.05) (Table 2). However, after completion of the WE program, energy expenditure in the training cohort was significantly increased compared with the control cohort. No differences were observed in these characteristics between participants who remained in the study and those who dropped out. The analysis presented below includes appropriate incorporation of non-compliance data.

**TABLE 1:** Baseline characteristics of participants of the control and WE groups.

	Control (*n* = 51)	WE (*n* = 60)
Age (year)	614	624
Sex (F/M)	41/10	50/10
Body mass (kg)	60.29.3	60.69.3
Height (cm)	155.96.2	156.16.3
Body mass index (kg/m^2^)	25.63.1	25.62.9
Body fat (%)	32.07.0	32.96.8
Fat mass (kg)	19.35.3	19.95.4
Fat free mass (kg)	40.96.4	40.76.9
Fat free mass (%)	69.07.9	67.06.9
**Circumference (cm)**		
Waist	82.08.9	82.58.9
Hip	97.27.0	97.76.8
Waist to hip circumference ratio	0.840.06	0.840.07

*Data are expressed as means ± SD. WE, wand stretching exercise.*

**TABLE 2:** Daily dietary intake and energy expenditure of the control and WE groups before and after 15 weeks.

Daily dietary intake and energy expenditure	Control (*n* = 51)		WE (*n* = 60)	
		
	Before	After	Before	After
Carbohydrate (g)	28710	26812	2449	23012
Fat (g)	513	534	503	463
Protein (g)	786	824	735	754
Dietary intake (kJ/day)	7,163238	7,075230	6,962246	6,815267^#^
Energy expenditure (kJ/day)	6,799192	6,832159	6,895213	7,155205**^+^**

*Data are expressed as means ± SE.*

*WE, wand stretching exercise.*

*^+^Significantly different from the control group after 15 weeks, *p* < 0.05.*

*^#^Significantly different from the energy expenditure after 15 weeks, *p* < 0.05.*

### Participants’ Compliance With the Exercise Program

Participants completing the study had higher than 90% compliance with the exercise training.

### Anthropometry and Body Composition

After completion of the WE program, the average value of WC for participants in the training cohort was significantly lower than that in the control cohort for both overweight (*d* = 0.48) and obese (*d* = 0.59) groups (both *P* < 0.01) (Tables 3, 4). Moreover, those who were overweight in the training cohort had a lower WC/HC ratio than those in the control group (*P* < 0.05, *d* = 0.30) (Table 3). Other anthropometry measurements showed no significant change in either cohort (Tables 3, 4). No significant effects of sex or age were observed on any of the parameters.

**TABLE 3:** Anthropometry, body composition, and BMR in participants with a BMI < 25 kg/m^2^ before and after 15 weeks in the control and WE groups.

	Control (*n* = 29)			WE (*n* = 27)			Mean difference (95% confidence interval)	*p*-value^b^	Hedges’
		
	Before	After	*p*-value^a^	Before	After	*p*-value^a^			
Weight (kg)	64.21.49	64.01.49	0.54	65.61.05	64.71.07	0.00	−0.66(−1.54*to*0.23)	0.14	0.14
BMI (kg/m^2^)	27.10.28	27.00.33	0.58	27.40.26	27.10.26	0.00	−0.24(−0.62*to*0.13)	0.19	0.30
BF%	35.41.29	34.21.38	0.25	34.61.27	32.91.22	0.00	−0.49(−2.16*to*1.77)	0.56	0.16
FM (kg)	22.70.97	21.91.09	0.28	22.60.83	21.20.82	0.00	−0.59(−1.85*to*0.66)	0.34	0.07
FFM (kg)	41.61.38	42.01.15	0.52	43.11.25	43.41.21	0.07	0.04(−0.91*to*0.99)	0.93	0.37
% FFM	67.33.00	68.64.00	0.34	66.13.00	68.42.00	0.0005	0.77(−1.70*to*3.20)	0.52	0.35
WC (cm)	86.91.63	87.41.65	0.35	87.00.91	85.01.00	0.0003	−2.58(−4.19*to*−0.97)	0.002	0.48
WC/HC	0.860.02	0.870.02	0.01	0.860.01	0.850.01	0.44	−0.02(−0.04*to*−0.004)	0.018	0.30
BMR (kJ/day)	59.82.05	56.32.65	0.07	58.21.87	63.01.73	0.006	7.82(2.99*to*12.65)	0.002	0.59
Fat oxidation rate (g/min)	0.050.01	0.070.01	0.08	0.060.002	0.070.002	0.60	0.021(−0.073*to*0.102)	0.17	0.11

*Data are expressed as means ± SE and mean differences of the subsequent measurement of each variable (adjusted by its baseline) (95% confidence interval); control group (*n* = 29, 22 females, 7 males) and WE group (*n* = 27, 23 females, 4 males). Analysis was performed using repeated measure analysis of variance (ANOVA). When a significant difference was observed, a *post hoc* analysis using the Bonferroni adjustment was performed.*

*^*a*^Test for significant differences within groups.*

*^*b*^Test for significant differences of subsequent measurements of each variable (adjusted by its baseline) between groups.*

*WE, wand stretching exercise; BMR, basal metabolic rate; BMI, body mass index; BM, body mass; BF, body fat; FM, fat mass; FFM, fat free mass; WC, waist circumference; WC/HC, waist to hip circumference ratio.*

**TABLE 4:** Anthropometry, body composition, and BMR in participants with BMI > 25 kg/m^2^ before and after 15 weeks in the control and WE groups.

	Control (*n* = 22)			WE (*n* = 33)			Mean difference (95% confidence interval)	*p*-value^*b*^	Hedges’
		
	Before	After	*p*-value^a^	Before	After	*p*-value^a^			
Weight (kg)	73.94.28	73.14.0	0.55	75.23.61	74.83.32	0.42	0.59(−3.77*to*4.96)	0.74	0.21
BMI (kg/m^2^)	31.70.49	31.40.38	0.57	32.20.78	32.10.65	0.42	0.45(−1.77*to*2.07)	0.51	0.22
BF%	38.22.51	38.02.48	0.76	36.95.78	36.45.91	0.08	−0.31(−2.58*to*1.97)	0.74	0.26
FM (kg)	28.01.66	27.51.47	0.64	27.74.41	27.24.39	0.12	−0.09(−3.33*to*3.16)	0.95	0.12
FFM (kg)	45.94.28	45.64.16	0.40	47.54.98	47.65.09	0.47	0.55(−0.86*to*1.97)	0.36	0.38
% FFM	62.01.0	64.01.0	0.10	62.00.8	64.00.7	0.0004	0.08(−1.50*to*1.60)	0.92	0.58
WC (cm)	87.01.4	87.31.2	0.57	86.91.0	84.71.0	0.0004	−2.5(−4.10*to*−0.90)	0.003	0.59
WC/HC	0.890.02	0.880.03	0.46	0.920.06	0.930.06	0.05	0.02(−0.05*to*0.09)	0.41	0.02
BMR (kJ/day)	58.84.09	62.56.41	0.50	72.712.84	81.810.46	0.13	7.57(−14.48*to*29.62)	0.42	0.61
Fat oxidation rate (g/min)	0.060.01	0.080.01	0.18	0.070.002	0.070.002	0.92	0.009(−0.127*to*0.132)	0.52	0.46

*Data are expressed as means ± SE and mean differences of the subsequent measurement of each variable (adjusted by its baseline) (95% confidence interval); control group (*n* = 22, 20 females, 2 males) and WE group (*n* = 33, 27 females, 6 males). Analysis was performed using repeated measure analysis of variance (ANOVA). When a significant difference was observed, a *post hoc* analysis using the Bonferroni adjustment was performed.*

*^*a*^Test for significant differences within groups.*

*^*b*^Test for significant differences of subsequent measurements of each variable (adjusted by its baseline) between groups.*

*WE, wand stretching exercise; BMR, basal metabolic rate; BMI, body mass index; BM, body mass; BF, body fat; FM, fat mass; FFM, fat free mass; WC, waist circumference; WC/HC, waist to hip circumference ratio.*

### Basal Metabolic Rate

The WE program induced a significant increase in BMR among the overweight participants alone, compared with the control group (*P* < 0.05, *d* = 0.59) (Table 3).

### Fat Oxidation Rate

Fat oxidation rates after 15 weeks of WE training and those in the control group showed no significant change in both overweight and obese groups (Tables 3, 4).

### Blood Chemistry Analysis

Compared with the control group, overweight participants in the WE group showed a tendency to reduced LDL-C levels (*P* = 0.09, *d* = 0.57, Table 5) and LDL-C/HDL-C ratio (*P* = 0.08, *d* = 0.37, Table 5). Moreover, within the overweight group, the WE showed significant improvement in the HDL-C, TC/HDL-C, LDL-C/HDL-C, and hsCRP levels after completion of the exercise program (all *P* < 0.05, Table 5). In addition, within the obese group, no significant differences were noted in any blood parameters after completion of the study (Table 6). For both the training and control cohorts, no differences were noted in any of the blood parameters between the BMI groups (Tables 5, 6).

**TABLE 5:** Blood chemistry parameters in participants with BMI < 25 kg/m^2^ before and after 15 weeks in the control and WE groups.

	Control (*n* = 29)			WE (*n* = 27)			Mean difference (95% confidence interval)	*p*-value^b^	Hedges’
		
	Before	After	*p*-value^a^	Before	After	*p*-value^a^			
FBG (mg/dL)	95.12.69	91.62.87	0.22	95.41.76	94.61.66	0.51	2.8(−2.1*to*7.6)	0.26	0.50
Insulin (uIU/mL)	10.60.66	10.40.66	0.94	14.80.34	12.30.26	0.16	−1.7(−14.1*to*14.9)	0.43	0.07
HOMA	3.90.49	6.12.31	0.32	3.70.33	3.90.34	0.49	−2.0(−5.5*to*1.4)	0.24	0.10
TG (mg/dL)	163.541.70	134.623.70	0.29	129.612.6	113.710.80	0.07	−4.0(−33.4*to*25.4)	0.79	0.37
TC (mg/dL)	224.97.94	222.97.62	0.84	215.55.81	205.85.35	0.07	−13.2(−29.9*to*3.6)	0.12	0.49
HDL-C, mg/dL)	57.42.70	59.63.19	0.37	55.32.57	59.62.68	0.02	1.7(−4.3*to*7.7)	0.57	0.19
LDL-C (mg/dL)	131.59.76	136.38.13	0.47	130.45.98	123.35.73	0.17	−12.4(−27.0*to*2.3)	0.09	0.57
TC/HDL-C	4.00.22	3.80.17	0.37	4.20.24	3.60.18	0.002	−0.3(−0.66*to*0.14)	0.19	0.11
LDL-C/HDL-C	2.30.16	2.30.16	0.75	2.50.17	2.20.85	0.006	−0.3(−0.58*to*0.04)	0.08	0.37
hsCRP (mg/L)	2.30.51	1.90.53	0.18	2.60.37	1.80.31	0.004	−0.3(−0.99*to*0.44)	0.44	0.03

*Data are expressed as means ± SE and mean differences of the subsequent measurement of each variable (adjusted by its baseline) (95% confidence interval); control group (*n* = 29, 22 females, 7 males) and WE group (*n* = 27, 23 females, 4 males). Analysis was performed using repeated measure analysis of variance (ANOVA). When a significant difference was observed, a post hoc analysis using the Bonferroni adjustment was performed. ^*a*^Test for significant differences within groups. ^*b*^Test for significant differences of subsequent measurements of each variable (adjusted by its baseline) between groups. WE, wand stretching exercise; FBG, fasting blood glucose; HOMA, homeostatic model assessment; TG, triglycerides; TC, total cholesterol; HDL-C, high-density lipoprotein cholesterol; LDL-C, low-density lipoprotein cholesterol; hsCRP, high-sensitivity C-reactive protein.*

**TABLE 6:** Blood chemistry parameters in participants with BMI > 25 kg/m^2^ before and after 15 weeks in the control and WE groups.

	Control (*n* = 22)			WE (*n* = 33)			Mean difference (95% confidence interval)	*p*-value^b^	Hedges’
		
	Before	After	*p*-value^a^	Before	After	*p*-value^a^			
FBG (mg/dL)	101.04.34	103.88.73	0.71	94.75.5	101.35.69	0.01	4.8(−23.5*to*33.0)	0.68	0.07
Insulin (uIU/mL)	18.20.40	17.50.4	0.81	15.90.2	16.90.2	0.55	0.33(−22.4*to*31.7)	0.61	0.38
HOMA	6.10.77	4.90.87	0.29	3.90.79	4.70.79	0.67	−0.13(−4.7*to*4.5)	0.95	0.21
TG (mg/dL)	145.434.4	139.429.8	0.61	133.016.5	97.711.9	0.27	−32.4(−87.0*to*22.3)	0.19	0.33
TC (mg/dL)	217.420.7	223.816.4	0.71	175.312.2	181.014.2	0.56	−19.7(−79.2*to*39.8)	0.44	1.11
HDL-C, mg/dL)	58.42.9	72.622.4	0.54	59.07.6	62.012.5	0.72	−11.8(−91.0*to*67.4)	0.72	0.14
LDL-C (mg/dL)	130.015.6	113.026.8	0.36	89.722.1	99.319.8	0.13	35.1(−36.2*to*106.8)	0.26	0.28
TC/HDL-C	3.70.33	3.90.67	0.79	3.10.55	3.20.83	0.76	0.37(−1.3*to*2.0)	0.59	0.24
LDL-C/HDL-C	2.20.26	2.20.58	0.97	1.70.54	1.90.72	0.49	0.51(−1.1*to*2.1)	0.45	0.15
hsCRP (mg/L)	3.41.14	3.91.28	0.37	5.12.34	4.02.41	0.30	−1.6(−4.2*to*0.99)	0.18	0.16

*Data are expressed as means ± SE and mean differences of the subsequent measurement of each variable (adjusted by its baseline) (95% confidence interval); control group (*n* = 22, 20 females, 2 males) and WE group (*n* = 33, 27 females, 6 males). Analysis was performed using repeated measure analysis of variance (ANOVA). When a significant difference was observed, a post hoc analysis using the Bonferroni adjustment was performed.*

*^*a*^Test for significant differences within groups.*

*^*b*^Test for significant differences of subsequent measurements of each variable (adjusted by its baseline) between groups.*

*WE, wand stretching exercise; FBG, fasting blood glucose; HOMA, homeostatic model assessment; TG, triglycerides; TC, total cholesterol; HDL-C, high-density lipoprotein cholesterol; LDL-C, low-density lipoprotein cholesterol; hsCRP, high-sensitivity C-reactive protein.*

## Discussion

This study proved that completion of a 15-week WE program can significantly reduce abdominal obesity and the WC/HC ratio, and increase BMR in sedentary Thai adults who are overweight. Furthermore, the WE program can reduce abdominal obesity in obese adults.

We hypothesized that the WE could reduce the CVRF, as measured by anthropometry, body composition, blood chemistry, BMR, and fat oxidation rate in healthy overweight or obese participants. Therefore, our findings of reduced WC in both groups, and reduced WC/HC ratio and increased BMR in the overweight group, partially confirms our hypothesis.

The WC measurement used in the present study was previously described in the WHO guidelines 2000 (the midpoint between the lower border of the rib cage and the iliac crest). It is accepted as a reliable, feasible measure of abdominal obesity (Rexrode et al., 2001; de Koning et al., 2007) that is convenient for both the practitioner and the general public. The WC has been used previously to assess CVRF in both men and women (Lois et al., 2008) because of the significant association. Thus, it is a useful indicator for exercise recommendations for this clinical population that can lead to reduced risks of future non-communicable diseases (Ortaglia et al., 2020).

Nevertheless, the WC has potential limitations compared with more direct or 3-dimensional measurements obtained using dual X-ray absorptiometry or axial computed tomography imaging (Makimura et al., 2008; Wiklund et al., 2008). Importantly, the coefficient of variation for the measurements of WC was 2.1%, which is acceptable. Thus, use of the WC is both reliable and valid to determine abdominal obesity. Previously however, the measurement protocol reportedly had no influence on any association between the WC and CVD mortality (Ross et al., 2008).

The WC can potentially provide sufficient evidence to confirm the beneficial effects of WE, as observed in the reduced CVRF among overweight and obese participants. Furthermore, the reduction in the WC/HC ratio, which is another potential indicator of abdominal obesity (World Health Organization. Regional Office for the Western Pacific, 2000) confirmed the beneficial effects of WE in reducing abdominal obesity in overweight participants. The reduction of the WC/HC ratio among obese participants may be due to reduced movement at the waist compared with the overweight participants (based on our observations during practice). The greater WC of obese participants made them move less than the overweight participants. Further investigations in a larger cohort and with a longer duration of training is highly desirable in the obese group.

The mechanism underlying the reduction in abdominal obesity in the present study has not been elucidated. One of the expected mechanisms is an exercise-induced increase in the fat oxidation rate, which occurs in muscle mitochondria and is induced by the enzymes citrate synthase and cytochrome C oxidase (Short et al., 2003). However, we observed no significant effects of WE training on the fat oxidation rate. One might argue that we did not measure this at the mitochondrial level. However, we indirectly measured fat oxidation by using the whole-body oxidation rate, as we collected expired gas. The indirect method used in the present study has been shown to be reliable and valid enough to measure the fat oxidation rate (Ghanassia et al., 2006; Prasertsri et al., 2013).

Another possible mechanism of the reduction in abdominal obesity is hydrocarbon redistribution. This hypothesis suggests a negative energy balance in fat cells due to the competition from skeletal muscle for circulating hydrocarbon sources (Kuo and Harris, 2016). The WE was moderate-intensity exercise, which has been shown to reduce abdominal fat (Rashti et al., 2019). However, this mechanism was not confirmed in this study, because muscle mass determined by the fat free mass was increased in the WE group alone without any significant differences in the control group.

A higher intensity WE may significantly improve the fat free mass. Furthermore, the WE under investigation in the present study comprises movements around the waist area, including flexion, extension, rotation, and side flexion. During these movements, all components of the WE involved movement at the waist, with frequent contraction and stretching of the abdominal and back muscles, and this may reduce the WC. This is supported by the study of Lahelma et al. (2019), who found that exercise using a weighted hula-hoop can reduce the WC in overweight subjects. This makes it particularly suited to reducing abdominal obesity. Either a higher intensity or longer duration can increase abdominal muscle mass.

The WE increased the BMR in the overweight participants alone. We do not yet know the reason for this phenomenon because the mass of skeletal muscle, an important tissue associated with the BMR, was not significantly increased. Thus, the increased BMR seems to be unrelated to any increase in muscle mass. This finding is consistent with a previous study that showed an increased BMR with an unchanged fat-free mass after endurance exercise training (Poehlman et al., 1990). They suggested that the variations in maximal oxygen consumption (*V̇*O_2*max*_) which is a significant predictor of resting metabolic rate (RMR) may contribute to individual variation in RMR in healthy older men. RMR is a more common measurement which uses less strict criteria than BMR (McMurray et al., 2014). This is comparable with our previous study that showed improved *V̇*O_2*max*_ determined by 6-min walk test according to WE training (Puengsuwan et al., 2008). The other explanation may be due to genetic effect since changes in RMR and *V̇*O_2*max*_ following the short-term exercise training are genotype dependent. However, the increase in BMR according to WE training could provide beneficial effect on stabilizing body composition to sedentary overweight people. This may permit us to intake higher energy without an increase in body fat mass. BMR is also affected by many factors such as menstrual cycle (Lawson et al., 1987). However, all female subjects in this study were in menopause. Therefore, the high BMR during the luteal phase were not found by this study. Furthermore, the increased BMR in this study may not be attributed to “carry over” effect of the last exercise bout because it was assessed at least 24 h after the last exercise bout. Even after the high-intensity exercise (15–48 h post-exercise), no changes in RMR were found (Devlin and Horton, 1986; Poehlman et al., 1989). Thus, after WE, moderate-intensity exercise, in this study it seems to have no “carry over” effect of the last exercise bout which took around 48 h after.

The WE training group had increased energy expenditure compared with the control group and higher energy intake within the group. This should cause a negative energy balance and should have resulted in body mass reduction. However, the greater energy expenditure in the WE group did not cause any reductions in body mass in either the overweight or obese groups, as compared with the control group. Furthermore, the WE training did not alter body composition, fat, or muscle mass. This is possibly due to our lack of restrictions on the daily diet of the participants. In addition, the difference between energy expenditure and energy intake was not large enough to result in any considerable negative energy balance. Thus, body composition was not significantly changed.

Notably, neither overweight nor obese participants showed significant changes in blood chemistry variables after exercise training. However, we found a tendency in the overweight group to have reduced LDL-C (*p* = 0.09) and LDL-C/HDL-C (*p* = 0.08), as compared with the control group. This seems to be consistent with the within-group results, as the WE group showed a significant increase in HDL-C concentration (*p* = 0.02), and reductions in TC/HDL-C (*p* = 0.002) and LDL-HDL-C (*p* = 0.006).

We used 12 weeks of training with 3-week pre-training to prevent injury from starting the WE. During the first 3 weeks, participants performed the WE during a 20-min session on each of three days. After the first 3 weeks, they performed the WE training by two 20-min sessions per day, 5 days per week for the next 12 weeks. At least 150 min per week of moderate-intensity aerobic training is recommended for weight reduction by ACSM (Donnelly et al., 2009). Importantly, several studies have demonstrated that 12 weeks of this exercise training program provided significant beneficial effects on obesity and cardiovascular risk factors such as decreased waist circumference (Saremi et al., 2010), body weight (Schjerve et al., 2008; Seo et al., 2011), fasting glucose level (Seo et al., 2011), lipid profiles (Schjerve et al., 2008; Seo et al., 2011), and diastolic blood pressure (Schjerve et al., 2008; Seo et al., 2011).

This study has several limitations. Firstly, most participants were female (the female vs. male ratio was 4.5 vs. 1) and abdominal obesity is more specific to the male population (Yoo et al., 2010). Therefore, the results of this study cannot be applied to the male population. Secondly, the duration of training may have been too short to improve visceral and subcutaneous fat and muscle compartments. Thirdly, specific assessments of the utilization of visceral and subcutaneous fat compartments, including magnetic resonance imaging, computed tomography, proton magnetic resonance spectroscopy, or muscle biopsy (Sabag et al., 2017), and fat biopsy (Riis et al., 2019) should be considered for more comprehensive information. Further study on either a longer exercise duration or dietary restrictions may help to determine the favorable effects of a WE on all relevant parameters among overweight and obese participants.

## Conclusion

We have shown that a 15-week WE program comprising 40 min exercises, 5 days per week, produced a significant reduction in WC in a cohort of sedentary middle-aged Thai adults and an increase in BMR in those participants classified as overweight at baseline. This reduction in abdominal obesity and improvement in basal energy expenditure imply a reduced risk of CVD. The WE program is an effective at-home fitness program that requires minimal equipment. It is simple and convenient to apply at home and suitable for the elderly, overweight, or other individuals who may not be so able to participate in commonly offered training programs.

## Data Availability Statement

The raw data supporting the conclusions of this article will be made available by the authors, without undue reservation. Requests to access these datasets should be directed to NL, naruemon@kku.ac.th.

## Ethics Statement

The studies involving human participants were reviewed and approved by the Ethics Committee of Khon Kaen University (HE480102) in accordance with the 1964 Helsinki Declaration. The patients/participants provided their written informed consent to participate in this study. Written informed consent was obtained from the individual(s) for the publication of any potentially identifiable images or data included in this article.

## Author Contributions

NL conceived the idea for the manuscript, agreed on content, and contributed to the writing and editing of the manuscript. PP collected and analyzed the data and drafted the manuscript. RN did the medical cover. PP, C-HK, RC, RN, and NL contributed to the editing of the manuscript and approved the final draft of the manuscript. All authors contributed to the article and approved the submitted version.

## Conflict of Interest

The authors declare that the research was conducted in the absence of any commercial or financial relationships that could be construed as a potential conflict of interest.

## Footnotes

1 https://www.stata.com/

## Supplementary Material

Supplementary Figure 1 Wand stretching exercise description.
