# Supplementary material for: Wand Stretching Exercise Decreases Abdominal Obesity Among Adults With High Body Mass Index Without Altering Fat Oxidation
Source: Front Physiol. 2020 Oct 29;11:565573. doi: 10.3389/fphys.2020.565573 (PMC7658093; doi:10.3389/fphys.2020.565573)
Supplement: Supplementary Figure 1 — Wand stretching exercise description. [file Data_Sheet_1.pdf]

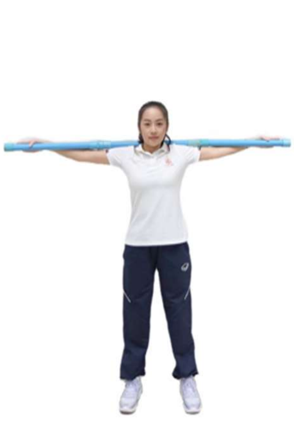

1) Shoulder abduction

Place a wand behind both shoulders. Both arms abduct to 90 degrees hanging on a wand. Raise one end of a wand toward a ceiling as high as possible while the other end pointing toward a floor. Return to starting position. Alternate both sides.

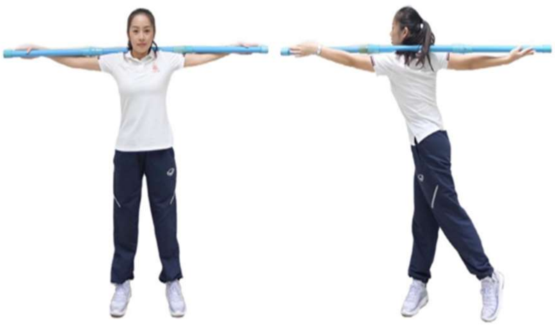

2) Body twisting

Do the same starting position as shoulder abduction exercise. Slowly twist a trunk toward one side as far as possible while bending the same knee with the other leg in a straight position. Return to starting position. Alternate both directions.

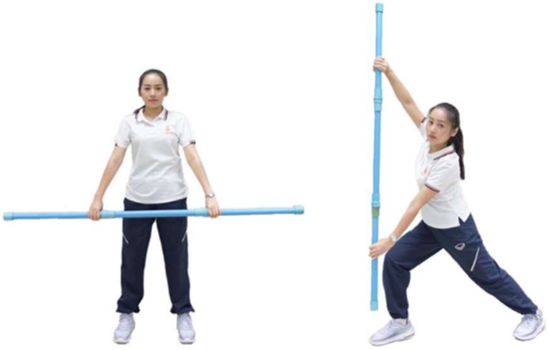

3) Lateral swing

Both hands hold a wand in front of thighs, and palms face forward. Shift body weight toward one leg with a bending knee while pointing that end of a wand toward ceiling until the arm touch the ear. Return to starting position. Alternate both directions.

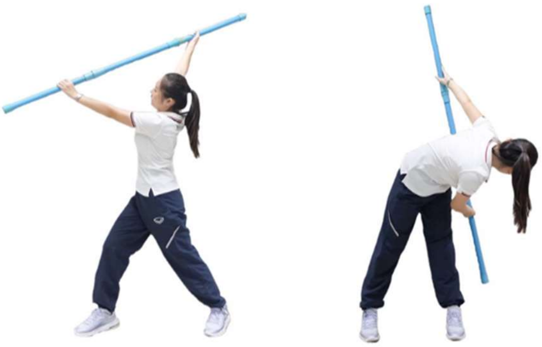

4) Rowing

Both hands hold a wand in front of thighs, and palms face a floor. Lift a wand over the head. Cycle a wand in a rowing style sideway as much as possible with a twisted trunk. Return to starting position. Alternate both sides.

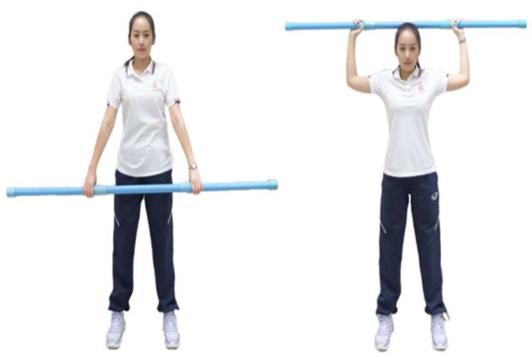

5) Lifting

Both hands hold a wand in front of thighs, palms face backward. Swing a wand over the head until resting on the back of the neck. Swing back to the starting position. Repeat the movements again.

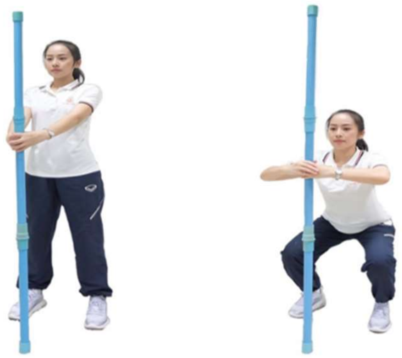

6) Sit down and stand up

Erect a wand on the floor just in front of a mid-position of both feet. Both hands grasp a wand at the shoulder level. Slowly bend both knees while keeping your back straight. Return to a starting position. Repeat the movements again.

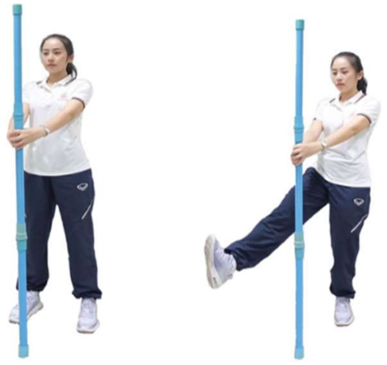

7) Kicking

Erect a wand on the floor just in front of a mid-position of both feet. Both hands hold a wand at the shoulder level. Shift body weight toward one leg. Slowly kick the other leg up front as high as possible. Keep the knee straight. Slowly return to a starting position. Alternate both legs.

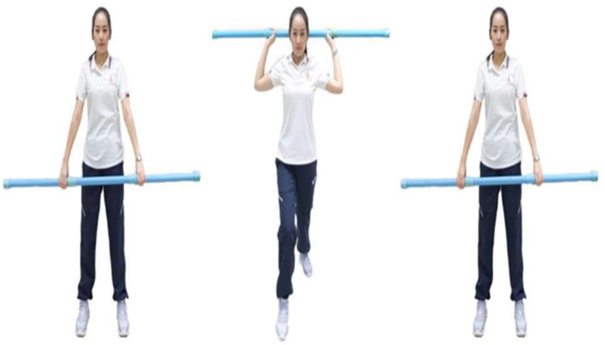

8) Lifting and bending

Both hands hold a wand in front of thighs, and palms face backward. Swing a wand over the head until resting on the back of the neck while bending both knees as low as possible. Swing a wand back to the starting position while straightening both knees. Repeat the movements again.

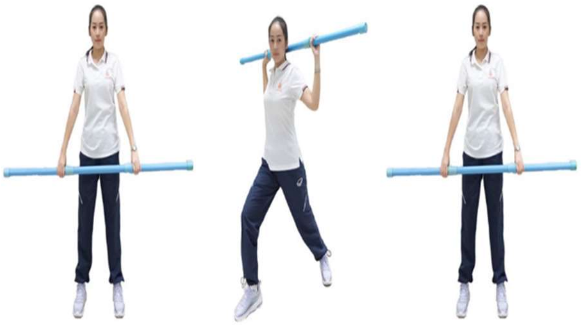

9) Stepping and lifting

Both hands hold a wand in front of thighs, palms face backward. Swing a wand over the head until resting on the back of the neck while stepping one leg forward with bending knee. Swing a wand back to the starting position while stepping back to the standing position. Alternate both legs.

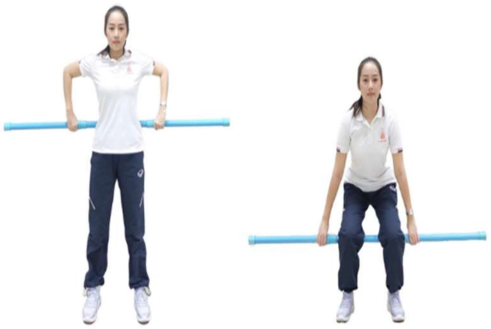

10) Back massaging

Both hands hold a wand behind lower back. Bend both knees no more than 90 degrees while rolling a wand up and down in a massaging way for 5 times. Return to a starting position. Repeat the movements

Supplementary Figure 1 Wand stretching exercise description
